# Induced Wettability Switch in Thin Films of Conductive Polymer Coatings Exhibiting Hydrophobic/Hydrophilic Interactions

**DOI:** 10.3390/s23156867

**Published:** 2023-08-02

**Authors:** Daniela Ionescu, Maria Kovaci

**Affiliations:** 1 Department of Telecommunications and Information Technologies, Faculty of Electronics, Telecommunications and Information Technologies, “Gh. Asachi” Technical University of Iasi, Carol I Blvd., No. 11, 700506 Iasi, Romania; 2 Department of Communications, Faculty of Electronics, Telecommunications and Information Technologies, “Politehnica” University of Timisoara, V. Pârvan Blvd., No. 2, 300223 Timisoara, Romania; maria.kovaci@upt.ro

**Keywords:** conductive polymer, interface, hydrophobic, hydrophilic, contact angle, dopant, structural simulation, wettability switch

## Abstract

The hydrophobic/hydrophilic character of some conductive polymer (CP) coatings can be switched in the function of the working conditions of these adaptive materials. We studied the influence of electrical stimuli and intrinsic physical characteristics (nature of the polymerizable core, dopants, the droplet dimension and physical properties, surface roughness, etc.) on the CP wettability. A simulation strategy was developed for determining the contact angle (CA) of a liquid droplet on a CP layer with roughness. The method was tested for new reported CP composites, but with new dopants. The results indicate that the influences on the material wettability are correlated, and in practice, modification of more than one parameter converges to a wanted behavior of the material. E.g., the CP porous film of poly(3-hexylthiophene) (P3HT) + [6,6]-phenyl-C61-butyricacid-methyl-ester (PCBM) changes its wettability at voltages of up to 26 V, but if doping ions are inserted and the roughness geometry is modified, the voltage decreases twice. Our multi-parametrical study points out that the polymer wettability type is driven by the voltage, but this effect is tuned differently by each internal parameter. The thin films’ effect and the dopants (in-situ and ex-situ) significantly decrease the actuation voltage. We also illustrated that the wettability type does not change for specific sets of parameters.

## 1. Introduction

New material technologies are using conductive polymers (CPs), exploited for the manufacture of coating layers presenting anion and cation exchange with the base material and for hydrophobic to hydrophilic interactions. Among the CP properties, catalyzing electrode reactions are important for electrochemical sensing, electrochemical supercapacitors, energy storage [1,2], etc. The color change of some CP combinations occurs under the influence of temperature or with the addition of oxide or alcohols and represents a property that is used to manufacture sensors or other devices as well [3]. Usually, silica is coated with polypyrrole or polypyrrolechloride because they present anion-exchange chromatographic behavior [4,5].

Hydrophobic CPs have non-polar molecules. A few examples, including new synthesized variants, are poly(3,4-ethylenedioxythiophene) polystyrene sulfonate (PEDOT:PSS) [6]; poly(3-hexylthiophene) + [6,6]-phenyl-C61-butyricacid-methyl-ester blend system (P3HT: PCBM) [7]; polypyrrole doped with perfluorooctanesulfonate (PPy-PFOS) [8]; etc.

Among the hydrophilic CPs, one can mention the catechol/quinone containing sulfonated lignin (LS) doped into various CPs, including polyaniline (PANI), polypyrrole (PPy), and poly(3,4-ethylenedioxythiophene) (PEDOT) [9]. 

An interesting characteristic of some composed CPs is hydrophobic/hydrophilic switching. Usually, surfaces with tunable properties respond to different stimuli, like electric, magnetic, optical, mechanical, or even environmental stimuli including temperature, humidity, and pH. Among the techniques applied for changing the CP wettability from hydrophobic to hydrophilic and vice versa, the literature has reported thermocapillarity, redox-active surfactants, optically reconfigured spiropyrans, or electrowetting [5]. The electrowetting method consists of applying a voltage with values of volts up to tens of volts on the polymer film, resulting in a charge accumulation near the three-phase liquid–solid–gas interface. This charge accumulation alters the balance of the interfacial surface tension forces and reduces the liquid contact angle (CA). This technique represents the most practical method for changing the CPs’ surface properties.

Different studies for achieving wettability switching by applying a voltage in a large interval (lower than 1 V and up to tens of volts) are reported in the literature, and the voltage value depends on different structural parameters [5,10,11,12,13]. A typical example is the polypyrrole (Ppy) [5,14], or new variants like the poly(1,4 cyclohexane isosorbide terephthalate)/polyvinyl alcohol [15], etc. This actuation voltage is lower for some materials and can be decreased when different parameters of the structure are modified. The phenomenon of lowering this voltage represents the task of our study. An example of a doped conductive polymer with a low actuation voltage is the dodecylbenzenesulfonate-doped polypyrrolePpy(DBS), which commutes its wettability from hydrophobic to hydrophilic in a redox process when an ultra-low voltage (~1 V) is applied due to the reorientation of the dopant molecules (DBS) in PPy [5,14]. 

We combined the mutual influence of different internal and external parameters in a simulation method in order to determine a controlled wettability switch that is necessary for specific applications. Usually, the experimental methods illustrate the evolution of the CA in the function of the actuation voltage in particular working conditions. The possibilities of varying an internal structural parameter while the voltage is varied are limited during experiments. For this reason, multiple-parameter analyses are not available in the literature. The actuation voltage’s dependences on more than one parameter in the same time can reveal new valences of the wettability commutation process, considering that this process is a threshold one. 

## 2. Materials and Methods

Physical properties of the conductive polymers can be fine-tuned via different methods like using dopants, changing the nature of the polymerizable core, using substituents [16], and controlling the synthesis method of the conductive polymer for varying the surface structure. Thus, their surface wettability can be adjusted. Surface effects can be observed, meaning a superhydrophilicity or superoleophobicity degree, characterized with the help of the contact angle formed by the tangent to the liquid drops’ free surface and the polymer layer in the contact point.

The combinations of materials considered in this study consist of a CP with different dopants, which facilitate the control of the surface wettability of the polymer. There are two types of important dopants for changing the polymer properties, including in-situ dopants, which are incorporated during the polymerization of monomers, and ex-situ dopants, which are inserted by the exchange of an ion in a pre-formed conductive polymer film with a different ion by cycling the electrode potential between oxidized and reduced forms of the conductive polymer [17,18,19,20]. Consequently, dopants like sodium dodecylbenzene sulfonate (DBS), dodecylbenzene sulfonic acid (DBSA), and benzenesulfonic acid (BSA), which decreases surface resistivity and increases the environmental stability, have been considered, but also anions/cations like BF_4_^−^, ClO_4_^−^, hexafluorophosphate (PF_6_)^−^, CF_3_SO_3_^−^/tetrabutylammonium (TBA^−^), tetraethylammonium(TEA^+^), andtetramethylammonium(TMA^+^), K⁺, NH₄⁺, and Mg_1/2_⁺ have been considered for tuning the contact angle. Dopants have been considered to be inserted in proper CP. The first tested CP was polypyrrole (Ppy) doped with anions, which is often reported in the literature, for verifying the viability of our simulation method. Then, the method was tested for doped Ppy(DBS) and then the method was applied for new variants of combinations such as polymer + dopants. Two examples of considered combinations are conductive polymer porous film with tunable wettability and adhesion fabricated by the chloroform solution of poly(3-hexylthiophene) (P3HT) and [6,6]-phenyl-C61-butyricacid-methyl-ester (PCBM) with water droplets [7,21] (denoted as P3HT:PCBM), and a polymer (PEDOT-PSS and polyethylenimine(PEI)) and fluoropolymer solution used to ensure a hydrophobic dielectric surface coating with electro-wetting in oil (denoted as PEDOT:PSS−PEI), respectively [22,23]. At the same time, biocompatible polymers represented materials of interest, like the polyvinyl alcohol/poly(1,4 cyclohexane isosorbide terephthalate) (PVA/PICT) blended through co-electrospinning [15], with dopant, which can change their surface wettability.

**Figure 1:**
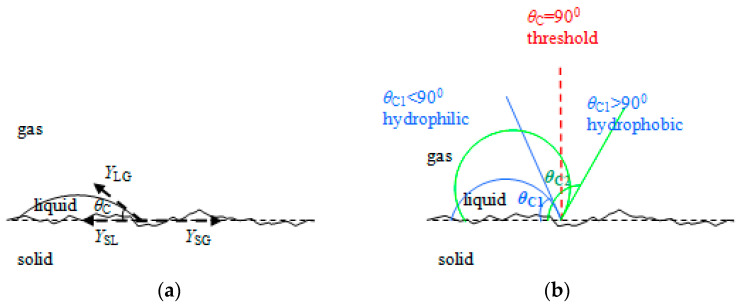
(**a**) A liquid droplet on a conductive polymer layer with roughness, with an illustration of the contact angle; (**b**) evolution of the contact angle from hydrophilic to hydrophobic near the threshold region (*θ_C threshold_* = 90°).

The contact angle in classical physics is characterized using Young’s equation, considering the adhesion/cohesion forces at the interfaces (see Figure 1). But at nanoscale, the line tension and Laplace pressure have to be considered, with the contact angle being indicated in this case by a new equation (Jasper, 2017) [5,11,12] as follows:

(1) $$\cos(\theta_{C})=\frac{\gamma_{SG}-\gamma_{SL}}{\gamma_{LG}}+\frac{\kappa }{\gamma_{LG}}\frac{1}{a}-\frac{\gamma }{3\gamma_{LG}}(2+\cos(\theta_{C})-2\cos^{2}(\theta_{C})-\cos^{3}(\theta_{C}))$$

where *γ*_SG_ is the interfacial tension between solid and gas, *γ*_SL_ is the interfacial tension between solid and liquid, *γ*_LG_ is the interfacial tension between liquid and gas, and *θ*_C_ is the contact angle at the interface. Coefficient *κ*[N] represents the line tension, and *a*[m] is the droplet radius; *γ* is the surface tension of the fluid’.

Due to the fact that the CA is strongly influenced by the surface roughness, the previous equation was corrected in agreement with the Wenzel correlation [7,14,24] as follows:

(2) $$\theta_{C\;real}=\mathrm{acos}(r\times \cos(\theta_{C}))$$

where *r* represents the roughness ratio, which is defined as the ratio between the real and projected (ideal flat) solid surface area (in our case, the CP layer).

When a voltage is applied at the droplet–solid interface, an electrical charge accumulates; there is a positive charge on the solid conductive polymer and a negative charge on the liquid face. The effect is a reduction in the effective interfacial tension *γ*_SL_, proportional to the surface charge density of the counter-ions on the liquid side [7]. Thus, the CP commutes its character from hydrophobic to hydrophilic and vice versa.

## 3. HFSS Set Up

A simulation strategy was developed in order to determine the CA of a liquid droplet on a conductive polymer layer with roughness.

The HFSS program (by Ansys) was used for structural simulation. The material description was performed at the molecular level. The molecular structures of different conductive polymers were implemented in a thin film with a thickness of 1000 nm. The liquid droplets (water) were deposited above, with diameter of 50 nm to 500 nm. In HFSS, the Volume of Fluid (VOF) model was the initial model used to describe the effect of surface tension along the interface between each pair of phases [25,26].

The interaction forces and, consequently, the surface tension between the different materials in contact, including the conductive polymer thin film, the liquid droplet (water), and gas (surrounded air), were determined. The result of these forces is tangent to the free surface of the droplet in the contact point with the solid layer, determining the contact angle (Figure 1).

**Figure 2:**
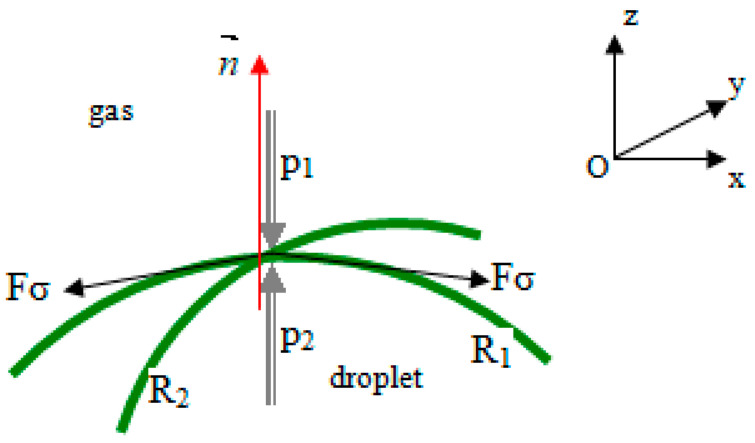
The interface between the liquid droplet and the surrounding gas. The surface tension arises as a result of attraction forces between molecules in a fluid.

The surface tension coefficient, *σ*, can be calculated using the following formula [25,27,28]:

(3) $$p_{2}-p_{1}=\sigma \left(\frac{1}{R_{1}}+\frac{1}{R_{2}}\right)$$

where *p*_1_ and *p*_2_ are the pressures exerted on the interface by the gas above and by the liquid below, respectively; *σ* is the surface tension coefficient; and *R*_1_ and *R*_2_ are the droplet surface curvatures as measured by two radii in orthogonal directions (Figure 2).

The surface normal at the liquid droplet next to the interface is given by [29] as follows:

(4) $$\hat{n}={\hat{n}}_{S}\cdot \cos(\theta_{C})+{\hat{t}}_{S}\cdot \sin\left(\theta_{C}\right)$$

where *θ_C_* represents the contact angle (CA) between the droplet and the polymer interface; ${\hat{n}}_{S}$ and ${\hat{t}}_{S}$ are the unit vectors normal and tangential to the solid polymer interface, respectively.

The combination of this contact angle with the surface normal calculated one cell away from the interface determines the local curvature of the droplet surface, and this curvature is used to adjust the body force term in the surface tension calculation [25,27].

Thus, the contact angle that the tangent at the fluid droplet is assumed to make with the polymer interface is used to adjust the surface normal in the cells near the interface. This dynamic boundary condition results in the adjustment of the curvature of the droplet surface near the interface [25,26,27,28,29,30,31,32].

In the second stage of the analysis, in ANSYS, we considered the continuum surface force (CSF) model, proposed by Brackbill et al. [24,29,30], for characterizing the surface tension phenomena. The liquid droplet surface curvature and the surface tension, respectively, were determined using the CSF model. Firstly, a verification was performed. The Reynolds number, *Re*, and the capillary number, *Ca*, were evaluated for the liquid at the polymer interface. Both of these quantities have to be much smaller than 1 in order for the surface tension effects to be consistent.

(5) $$Re=\frac{\rho uL}{\mu };\;\;\;\;Ca=\frac{\mu u}{\sigma }$$

where *ρ* is the fluid density; *u* is the flow speed (the free–stream velocity); *L* is a characteristic linear dimension; and *μ* is the dynamic viscosity of the fluid.

Then, in the simulation program, the **Phase Interaction Panel** was activated.

The CSF model enables a more accurate modeling of the three-dimensional fluid flow driven by the surface forces. The CSF model interprets the surface tension as a continuous, three-dimensional effect across an interface, rather than a boundary condition on the interface. The CSF model is a non-conservative model in which the surface forces are converted to volumetric forces, which is proper for describing the field forces in the matrix of polymeric chains. The surface curvature of the liquid droplet, *κ*, is calculated in terms of the divergence of the unit normal at the droplet free surface, $\hat{n}$ [32] as follows:

(6) $$\kappa =\nabla \cdot \hat{n};\;\;\;\;\vec{n}=\nabla \alpha_{i}$$

where $\hat{n}=\vec{n}/\left|n\right|$ is the unit normal corresponding to the surface normal $\vec{n}$, and *α_i_* is the volume fraction of the phase number *i* (liquid droplet here).

The volume force is calculated on the basis of the momentum equation for the two phases in contact (liquid droplet and gas, in Figure 2) as follows:

(7) $$F_{vol\;CSF}=\sigma_{ij}\cdot \frac{\rho_{ij}\kappa_{i}\cdot \nabla \alpha_{i}}{\frac{1}{2}\cdot \left(\rho_{i}+\rho_{j}\right)}$$

where σ*_ij_* is the surface tension between the two phases that are in contact, which has to be determined; $\rho_{ij}=\underset{i}{\sum }\alpha_{i}\cdot \rho_{i}$ is the volume-averaged density; $\rho_{i}$ and $\rho_{j}$ are the densities of the two phases; the curvatures are: $\kappa_{i}=-\kappa_{j}$; and the surfaces normal are: $\nabla \alpha_{i}=-\nabla \alpha_{j}$.

The calculation of the field forces (electric/gravitational/dissipative forces) was performed on the basis of the molecular model of each phase. Web service calculators, like MolCalc, the Socratic Q&A Calculator for Intermolecular Forces, etc., have been used in combination with the theoretical formula [33,34,35]. For the calculation of the interaction forces inter-system and intra-system in the polymer–liquid–gas structure, we had to consider the polymer structure, the chain configuration, how the in-situ and ex-situ dopants are integrated in the CP structure, and how the dopants interact with the polymeric chain; we also had to consider the liquid nature and the gravitational and dissipative effects. All of these parameters influence the result, and their influence is correlated; consequently, the wettability of the solid polymers can be changed when modifications are induced at the structural level.

The working strategy in HFSS consists of the following four main steps: modeling the free surface phenomena based on the VOF model equations; solving the VOF equations and determining the surface tension for the droplet placed on the solid polymer; setting the boundary conditions; solver settings and solving process optimization.

In the **Multiphase model** menu (from the **Physics** tab), the **Homogeneous Model** was selected as the “Volume of Fluid”. The **Interface Modeling** was set to “Sharp”. The Volume Fraction Cutoff was considered of 10^−8^ and the Courant Number of 0.2. For the **Setup** settings, the **Solver** was chosen “Pressure-based”, working in “Transient” regime. The gravitation forces have been considered.

At the **Volume Fraction Parameters**, the Formulation was set for an “Implicit” scheme, for which variable time stepping is available. The **Body Forces Formulation** was also set on “Implicit Body Forces” in order to have the possibility to include interaction forces between the solid polymer and the liquid droplet.

Then, the parameters for the **Coupled VOF Solver** were set to work with the following **Solution Methods**: **Pressure**–**Velocity Coupling** was: “Coupled with Volume Fractions”; solving process was with **Spatial Discretization**, with **Momentum** set on “Second Order Upwing” in a “Pseudo Transient” regime. For **Solution Controls,** a **VolumeFraction Courant Number** of a few hundred orders was considered until the results were reproducible.

In the HFSS simulation, a moving surface mesh was considered, with dimensions in the interval 10^−8^–10^−9^ m, correlated with the dynamic of the polymeric chain matrix with dopants. The considered mesh was polyhedral in order to cover the thickness of the different polymeric chains, at the level of the minimum dimension cell. This mesh is suitable with the considered model “interfacial anti–diffusion” and for the case of sharp interfaces. A high aspect ratio has to be reached. In some cases, for polymers with both in-situ and ex-situ dopants, a cut-cell mesh was used.

The adaptive time stepping was applied at solver settings, with a minimum step size of 10^−9^ s and maximum step size of 10^−6^ s. The time step can be estimated as follows: $\Delta t=\frac{\sqrt[{3}]{V_{cellmin}}}{u}$, where $V_{cellmin}$ is the minimum volume of the discretization cell. The time step was decreased until the results were reproducible. The time step was varied from one structure to another, with the solution convergence depending on each set of structural forces.

The adaptation control was activated in variant with the dynamic adaptation and advanced controls. The advanced stabilization method was also checked for simulation. The boundary conditions were carefully set in order to describe, with accuracy, the interface behavior of the conductive polymer.

In the **Wall Adhesion** menu, the “mixture” **Phase** was set for the “fluid” **Adjacent Cell Zone**. The “**Momentum**” analysis was performed with the “stationary wall”, and their **Motion** “Relative to Adjacent Cell Zone” was checked. In the submenu for **Wall Roughness**, the **Roughness Constant** value was introduced. The CA value of 90 ^0^ was set as a reference, for the system “water–liquid” in “air”, then for “oil–liquid” in air for different CPs.

Due to the fact that the curvature calculation at the droplet surface is based on volume fraction fields, to avoid convergence issues in the determination of the surface tension, we used node-based smoothening of the VOF field for the curvature calculation. The parameters for the node-based smoothing were the following: 2 (number of smoothing); 0.9 (smoothing relaxation factor). We used the VOF gradients at nodes for curvature calculation.

## 4. Results for the Variable CA

Our task was to commute the hydrophobic/hydrophilic character of a conductive polymer (CP) coating in the function of the working conditions of these adaptive materials. Generally, the electrical stimuli and intrinsic physical properties contribute to the change in the contact angle (CA) of a liquid droplet on the polymer surface. We studied the influence of the doping ion characteristics, the nature of the polymerizable core, and the roughness geometry on the wetting properties of the CP. The threshold behavior of the CP was monitored, and the induced CA switching possibilities were identified.

**Figure 3:**
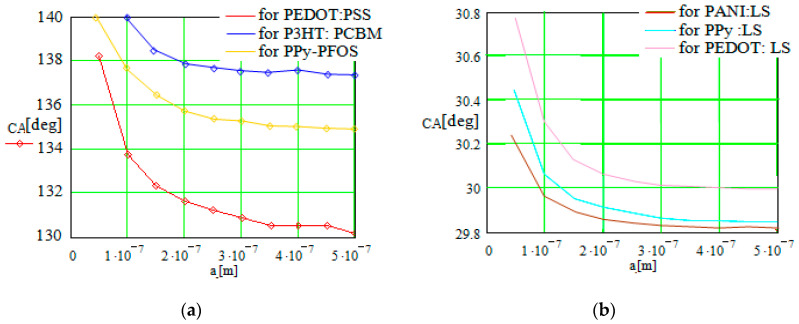
Contact angle variation in function of the droplet radius for (**a**) hydrophobic and (**b**) hydrophilic CP surfaces, respectively.

First, the method was applied to study the CA dependence on different parameters like the roughness degree of the conductive polymer layer, the droplet radius, or the surface tension of the fluid. A few sets of results for the hydrophobic and hydrophilic CPs with dopants are illustrated in Figure 3. The hydrophobic CP variants were PEDOT:PSS; P3HT:PCBM; and PPy–PFOS. The hydrophilic variants were PANI:LS; PPy:LS; and PEDOT:LS.

The contact angle is modified when different parameters are varied; thus, the surface wettability was adjusted by modifying the following material parameters: the nature of the polymerizable core, using substituents; the CP surface roughness; the radii of the liquid droplets; and the surface tension of the fluid (different types of liquid droplets were considered).

The Jasper equation, (1) being self-consistent (variable cos(*θ_C_*) depends by itself inside the expression), does not generate a function that can be easy represented on a theoretical graph. Consequently, an alternative method (a simulation in our case) is necessary in order to describe the contact angle evolution with the parameters included in the equation. The calculation strategy for the contact angle was based on implementing the VOF model in Mathcad [31] using the data given by the simulation.

We first analyzed the CA variation using the radii of the liquid droplets, for droplets with a radius of 50 nm to 500 nm. The interface between the conductive polymer layer and the liquid droplet was set to flat (*r* = 1). For the hydrophobic CP category, the results are indicated in Figure 3a. Figure 3b illustrates the CA evolution with the droplet radius in the case of the hydrophilic CP category.

The graphs indicate that if the droplet radius increases, the CA decreases, with larger droplets being strongly influenced by gravity (because they are heavier). The CA decrease is almost an exponential one, indicating a nonlinear dependence, which can be exploited in practice when the droplets are small and easier to control on the hydrophobic surface (can be easily directed by a force, the CA, with their adherence being poorer). At the same time, the droplet radius influence on the CA does not generate a large variation of this quantity, especially in the hydrophilic CP surfaces, in comparison with the parameters considered below.

**Figure 4:**
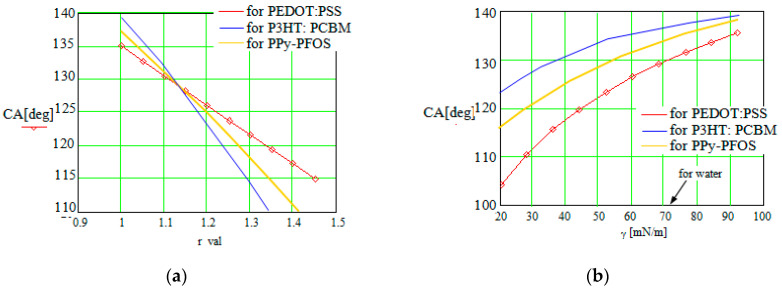
(**a**) Contact angle variation in function of roughness degree of the CP layer, hydrophobic type, for a droplet radius of 150 nm; (**b**) contact angle variation in function of the surface tension of the fluid. The hydrophobic CP case was considered. The roughness ratio is *r* = 1.25 for a droplet radius of 150 nm.

A surface with roughness was considered because the roughness influence was demonstrated to be significant in practice [7,16]. The drop dimensions are large in comparison with the scale of the roughness. For a roughness ratio, *r*, of up to 1.4, the simulation analysis was performed. The results in the case of the hydrophobic CP surfaces are illustrated in Figure 4a.

The results indicate an almost linear and abrupt decrease in the CA when the surface roughness increases. In practice, a rough surface has the tendency to absorb the droplet fluid in the interstices, with its shape becoming more flat. In the case of the hydrophobic conductive polymers with a higher CA close to 90°, the CP surface character can be changed to hydrophilic, but close to the threshold, by an increasing CP surface roughness.

The contact angle dependence on the surface tension of the fluid (droplet) is illustrated in Figure 4b. A rough surface was considered for the conductive polymer. The results are also indicated for the hydrophobic CP case. On the Ox axis, one remarks the *γ* value for water (surface tension coefficient of water is about 72 mN/m at room temperature). The surface tension of the fluid droplet can be modified in practice by adding different surfactants in the wetting water (or oil), e.g., dodecane (*γ*~25 mN/m) [5], or by using an artificial surfactant droplet, e.g., superhydrophobic polystyrene (PS) nanotube film [7], organic droplet (dichloromethane), etc. [22].

One observes that an increase in the surface tension of the fluid also increases the CA due to the fact that the fluid is more dense and wets the weaker solid surface. The influence of the internal forces in the liquid state (droplet) is also a strong one, if we consider the interval of the CA variation in comparison with the previous graphs.

The simulation method, in agreement with the theory, points out that all of these influences on the contact angle are correlated. By changing the different parameters of the system CP layer-wetting fluid, the optimal physical structure of the pairs can be chosen in order to obtain the desired value for the wettability.

Also, the character of the surface layer constructed of conductive polymers can be changed. Usually, in practice, this can be achieved by varying the contact angle in the function of an applied voltage (up to 20 V), and for different dopant levels. The simulation study demonstrated that, by correlating the solid–fluid system parameters, a transition from a hydrophobic to hydrophilic character of the solid surface can be achieved.

Our results are in agreement with the results reported in the literature for the parameters that characterize the hydrophobic/hydrophilic CPs [6,7,8,9].

After studying the contact angle dependence on different parameters, the simulation method was applied for determining the CA values when different CP composites behave in the threshold region, evolving between the hydrophobic and hydrophilic characteristics. The simulation method was applied for some new CP composites reported in the literature, but with diversified dopants, and the complex structure of each material variant was considered in detail. The CP surface characteristics were driven here by an electrical external stimulus, represented by an applied voltage.

The CP surface wettability was modified by changing different internal parameters at the same time. CPs are flexible structures, which easily incorporate dopants in-situ or ex-situ, with different roles, like modifying the CP conductivity, modifying the surface properties, and functionalizing and catalyzing different functions of the interface. It is also interesting that these capabilities of changing the CP’s physical properties, in particular, its intrinsic hydrophobicity, are reversible in many cases. The internal parameters modified in our study were the doping ion characteristics, the nature of the polymerizable core, and the roughness geometry of the CP interface.

**Figure 5:**
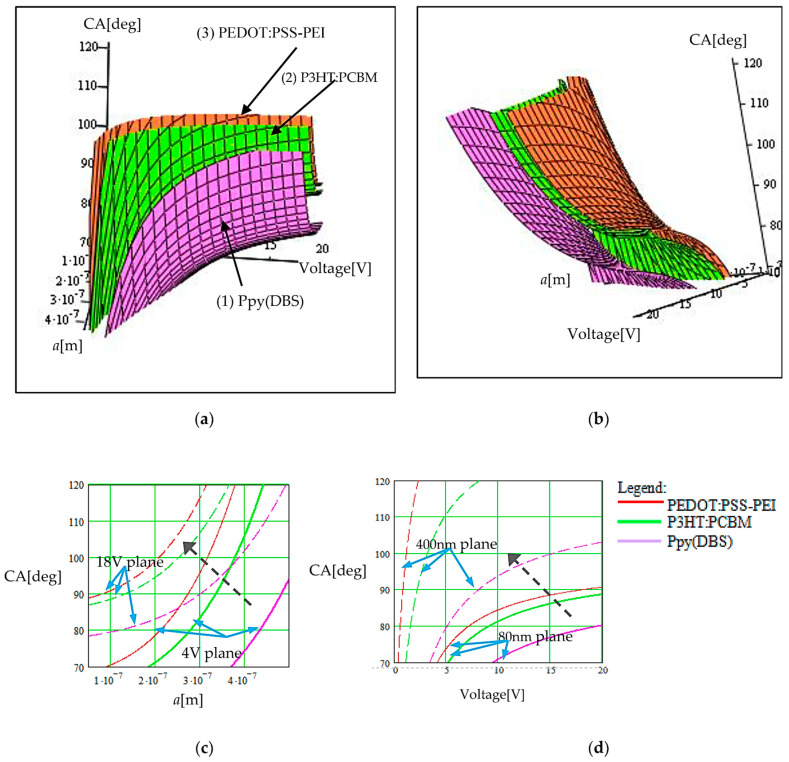
Illustration of the voltage-driven wettability modification from hydrophobic to hydrophilic and vice versa, when the droplet radius, *a*, is also changed. Different composed CPs were considered with dopants. (**a**) Graph view for observing the droplet radius values; (**b**) graph view for observing the applied voltage values; (**c**) cross-section in the 3D graph for constant voltages of 4 V (curves with continuous line) and 18 V (curves with dashed line); (**d**) cross-section in the 3D graph for constant droplet radius of 80 nm (curves with continuous line) and 400 nm (curves with dashed line). The black dashed arrow indicates how the plan for a constant parameter moves when that parameter increases.

**Figure 6:**
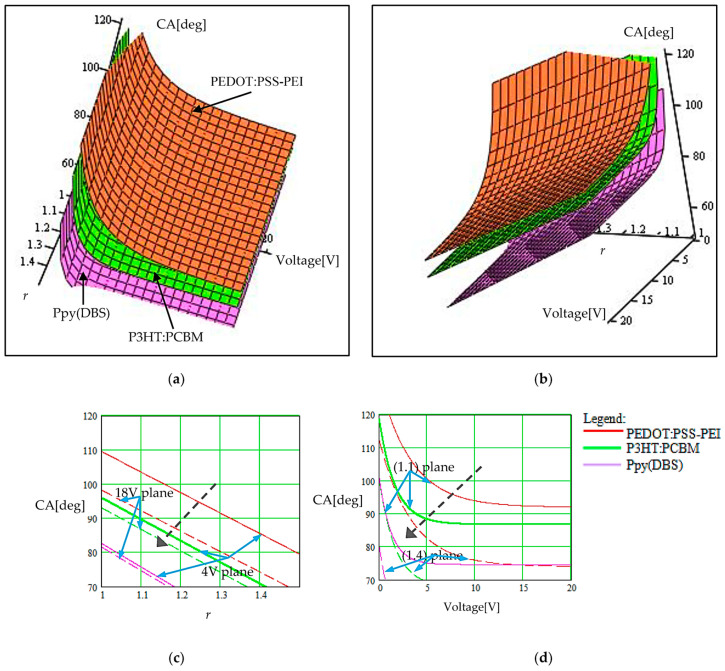
Illustration of the voltage-driven wettability modification from hydrophobic to hydrophilic and vice versa, when the roughness degree of the CP layer, *r*, is also changed. The droplet radius was 150 nm. (**a**) Graph view for observing the roughness degree values; (**b**) graph view for observing the applied voltage values; (**c**) cross-section in the 3D graph for constant voltages of 4 V (curves with continuous line) and 18 V (curves with dashed line); (**d**) cross-section in the 3D graph for constant roughness degree for *r* = 1.1 (curves with continuous line) and for *r* = 1.4 (curves with dashed line). The black dashed arrow indicates how the plan for a constant parameter moves when that parameter increases.

**Figure 7:**
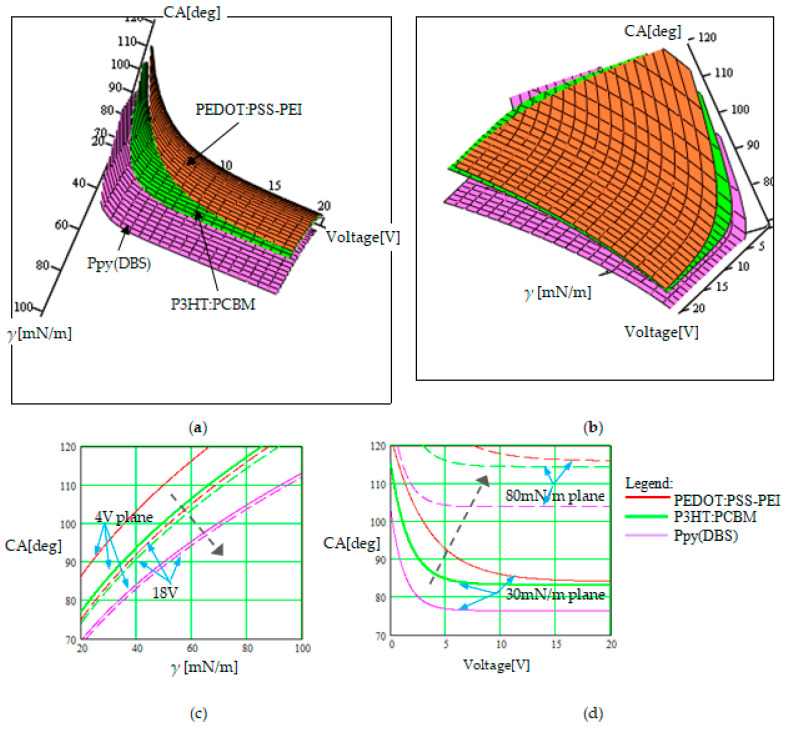
Illustration of the voltage-driven wettability modification from hydrophobic to hydrophilic and vice versa, when the surface tension of the fluid, *γ*, is also changed. The roughness ratio is *r* = 1.25 for a droplet radius of 150 nm. (**a**) Graph view for observing the surface tension values; (**b**) graph view for observing the applied voltage values. (**c**) Cross-section in the 3D graph for constant voltages of 4 V (curves with continuous line) and 18 V (curves with dashed line); (**d**) cross-section in the 3D graph for constant surface tensions of 30 mN/m (curves with continuous line) and 80 mN/m (curves with dashed line). The black dashed arrow indicates how the plan for a constant parameter moves when that parameter increases.

Double-parametrical graphs of the CA when the wettability character changes from hydrophobic to hydrophilic and vice versa, in the function of the applied electric voltage, considering a second variation of a previously specified internal parameter, are illustrated in Figure 5, Figure 6 and Figure 7. We considered, for exemplification, the cases of the following three types of doped CPs: Ppy(DBS) (denoted as CP(1)) (as reference material for verifying the method) [14,19], P3HT:PCBM (denoted as CP(2)), and PEDOT:PSS–PEI (denoted as CP(3)).

In Figure 5, we illustrate the dependence of the contact angle on the droplet radius, *a*, for different applied voltages for commuting the polymer wettability. Two views of the double-parametrical graph are presented in Figure 5a,b for observing the droplet radius values and the applied voltage values, respectively on the graph scales. Also, two cross-sections of the 3D graph are available for observing the CA evolution with an individual parameter when the other parameter is constant. In practice, these parameters are interdependent, and we considered this mutual influence in our analysis method. The first cross-section given in Figure 5c presents the evolution of the CA with the droplet radius when the applied voltage is constant. Two planes are represented for a smaller applied voltage and for a higher applied voltage in the considered interval (0–20 V), namely for 4 V (the continuous line curves) and 18 V (the dash line curves). The second cross-section given in Figure 5d presents the evolution of the CA with the applied voltage when the droplet radius is constant. Again, two planes are represented, containing the curves for the CA evolution when the droplet radius is 80 nm (continuous curves) and 400 nm (dash curves).

In each plane, the three curves correspond to the three polymers considered for analyses (red/pink for PEDOT:PSS−PEI; green for P3HT:PCBM; and mauve for Ppy(DBS)). In the 2D graphs, we indicated with a black dashed arrow how the plan for a constant parameter moves when that parameter increases.

The case of the anion dopants (NH₄⁺) is illustrated in the presented graphs, with the dopants inserted ex-situ. The influence of the in-situ dopants is higher regarding the CP conductivity, but the main influence on the surface tensions at the polymer interfaces is due to the ex-situ ions, anions, and cations. In comparison with the cases without dopants, modifications of up to 12% were encountered considering the contact angle variation.

The same decomposition strategy for the 3D graphs was applied in the case of the double-parametrical representations of the contact angle given in Figure 6 and Figure 7. In Figure 6, we represent the dependence of the contact angle on the roughness degree of the CP layer, r, when the applied voltage for commuting the polymer wettability was varied. Cross-sections of the 3D graph are available in Figure 6c,d for the cases of constant voltage and constant roughness degree, respectively. The cross-section plane was moved in two positions in each case, for a lower and higher value of the constant parameter. The obtained curves are indicated on the two 2D graphs (two sets of curves, continuous and dashed curves, respectively). Each set corresponds again to the three considered polymers (Ppy(DBS), P3HT:PCBM, and PEDOT:PSS–PEI).

Figure 7 illustrates the contact angle dependence on the surface tension of the fluid, γ, when a voltage in the interval (0–20 V) was applied to change the polymer wettability from hydrophobic to hydrophilic and vice versa. The 3D graph was rotated in two positions (like in Figure 5 and Figure 6 presented above) for visibility, in Figure 7a,b. The cross-sections of the 3D graph are represented for two constant voltage planes (Figure 7c) and for two constant γ planes (Figure 7d). The planes corresponding to the constant voltage migrate in the right-down direction when the voltage increases, and to obtain the same value for the CA, the surface tension of the fluid has to be increased. The planes corresponding to constant γ migrate to the right-up direction when γ increases, and to obtain the same value for the CA, the applied voltage also has to be increased. In each set of curves represented for a constant parameter, the three curves corresponding to the three analyzed polymers can be identified, like in Figure 5 and Figure 6 presented above.

For the CPs that can change their wettability, different methods of analysis have been reported in the literature. A comparative analysis was presented in order to reveal the advantages of our method of study. We considered for illustration the three polymers analyzed in the 3D parametrical graphs, Ppy(DBS), P3HT:PCBM, and PEDOT:PSS–PEI. The comparative analysis is given in Table 1. In the table, we present the contact angle evolution when an actuation voltage is applied in a specific interval, imposed by the doped polymer nature. In this interval, the wettability of the polymer changes or is intended to be changed, but this property is tuned by different control parameters. These control parameters are enumerated in the table, specifically for each analyzing method. One observes that our method offers the possibility of changing a greater number of parameters and allows for their influence to be monitored simultaneously.

## 5. Discussion

Our results indicate the following recommendations in order to modify the contact angle of a liquid droplet on different CP surfaces. By increasing the droplet radius, *a* (Figure 3), the CA decreases, meaning that the hydrophilic character of the CP surface is favored. The droplet dimensions are not recommended to be larger than a limit of a few hundred nm, correlated with the polymer macro-molecule dimensions, considering also that the CA decreasing effect is insignificant over a dimensional limit of the droplet radius.

When the surface roughness increases (Figure 4a), the CA decreases significantly. The effect is significant, and the CA decrease is almost linear, but it is not recommended for the surface roughness degree to overlap a value of 1.3–1.4 to avoid other unwanted effects due to supplementary particle interactionsat the interface.

For the wetting fluids with an increased surface tension (Figure 4b), the contact angle also increases, with the hydrophobic character of the CP surface being favored. In the case of water, which represents a wetting substance of interest in practice, its value of superficial tension of about 72 mN/m ensures a variation of more than 20% of the contact angle, correlated with the modification of the other parameters of the system.

Concerning the optimization process of changing the wettability of the doped CP from hydrophobic to hydrophilic, the double-parametrical graphs indicate that for a smaller droplet radius (Figure 5), the actuation voltage necessary for wettability commutation is higher and decreases more than twice when the droplet radius increases by up to ten times (50 nm to 500 nm). This is due to the fact that for smaller droplets, the internal tensions are lower, corresponding to a smaller amount of liquid substance and a lower number of interacting molecules of the same type, but at the same time, the gravitation forces decrease and do not favor the hydrophilic interactions with the polymer. Consequently, when the droplet dimensions are increasing, the wettability commutes to a hydrophilic character at lower voltages.

The roughness degree of the CP layer also influences the voltage applied on the CP for commuting the wettability. The higher the interface roughness (Figure 6), the lower the applied voltage for commutation. The phenomenon of increasing the adhesion forces at the interface level can be correlated with this effect.

When the surface tension of the fluid increases (Figure 7), it is necessary for the actuation voltage for the wettability commutation to be higher due to the fact that the report of adhesion/cohesion forces changes. This phenomenon is also dependent on the pair of phases characterized by mutual interaction, and consequently, the nature of the in-situ dopants strongly influences this effect.

The most important fact illustrated by our method is that the contact angle evolution is mainly driven by the applied voltage when the polymer wettability commutes, but the CA dependence on this voltage is strongly influenced by another parameter, which can be changed for wettability control. If we compare the 2D graphs illustrated in Figure 5d, Figure 6d, and Figure 7d, we observe an induced CA evolution in the function of the applied voltage, which is different when we modify a structural parameter or another. This can be explained when we analyze the molecular structure and the equilibrium of forces in the stable state of the polymer–droplet–gas system. Due to the flexibility of the long polymeric chains, it is important which structural parameter is changed when we want to determine the necessary voltage applied for wettability commutation. A matrix of polymeric chains with a particular configuration generates an amount of interaction forces at the surface, and this particular configuration is dynamic (long polymeric chains’ relative positions is variable). The chains’ relative positions depend on their mutual interaction forces, but near the surface, they are also influenced by interaction forces with the media in contact with the polymer.

The internal forces’ equilibrium at the interface implies that there are either molecular interaction forces, electric forces, or gravitational forces. The nature of the polymer and the liquid droplet are important for deciding the molecular interactions, but for a complete description of the field forces, we have to include the electric and gravitational forces at the macroscopic level. Geometrical parameters like the droplet radius and the roughness degree of the CP layer have to be considered because they help us to characterize the influence of these macroscopic forces.

Consequently, the results obtained via simulation illustrated in Figure 5d, Figure 6d and Figure 7d help us to identify different conditions necessary for changing the wettability of the polymer. The analyzed polymers change their wetting character from hydrophilic to hydrophobic (the CA increases from values lower than 90° to values higher than 90°) if the applied voltage increases when we modify the droplet radius. The effect is dominated by the value and distribution of the electric forces in comparison with gravitational forces in the field, as we have mentioned above. If we want to apply a smaller actuation voltage, a higher number of droplets with a higher radius are necessary. When the polymer surface roughness is modified, the wettability commutation occurs from hydrophobic to hydrophilic if we increase the voltage. A higher roughness favors the hydrophilic character (the real contact solid–liquid surface is increased by roughness, and consequently, the interaction forces are increased) and the actuation voltage is smaller. Finally, the polymer wettability also commutes from hydrophobic to hydrophilic by increasing the applied voltage when we modify the surface tension of the fluid. Liquids with a high surface tension have the tendency to be hydrophobic and, in many cases, do not commute to hydrophilic by applying a voltage because their internal forces are high in comparison with the other interaction forces at the interface.

The graphs illustrate another aspect that has to be considered in practice, namely that no wettability commutation occurs in some cases, for some internal and external parameter correlation. E.g., (1) in Figure 5d (the continuous mauve curve), we can observe that the Ppy(DBS) polymer covered with droplets of a small radius (80 nm) remains hydrophilic at applied voltages of up to 20 V. Higher voltages are not justified to be applied for efficiency reasons. (2) In Figure 6d (the continuous red curve), we can observe that the PEDOT:PSS–PEI remains hydrophobic at the same applied voltages of up to 20 V if the polymer surface roughness is insignificant (*r* = 1.1, with a small value). (3) In Figure 7d (the dash green curve), we can observe that the third considered polymer P3HT:PCBMremains hydrophobic at applied voltages of up to 20 V if the surface tension of the fluid is high (80 mN/m).

We have to point out another important property of the CPs, namely that all wettability modifications, induced by different parameter variations, are reversible. The simulation procedure was applied in order to determine the CA and its modifications when the polymer surface evolved from the hydrophilic to hydrophobic state and vice versa. Consequently, the CA evolution was monitored when its values were increasing and decreasing in respect with the threshold value of 90°. The obtained results are reproducible.

## 6. Conclusions

New synthesized conductive polymer structures, like PPy−PFOS or PEDOT: LS or P3HT:PCBM,were considered in this paper in order to analyze their hydrophobic/hydrophilic properties and the wettability change. The theoretical considerations were based on the CSF model (Brackbill et al. [29]), improved by Jasper et al. [5]. A simulation study was performed using the HFSS (Ansys) and Mathcad (PTC) programs, based on the VOF model and CSF models for the multi-phase polymer–liquid droplet–gas system.

The wettability switch of the composed conductive polymers having in-situ and ex-situ dopants is of great interest in practice for interface adaptabilities. For wettability switching from hydrophobic to hydrophilic and vice versa, the electrowetting procedure was considered, which was identified as an efficient method in the case of the analyzed structures. We combined a variation of different parameters like the nature of the polymerizable core, doping ion characteristics, droplet physical parameters, and roughness geometry, with an external voltage variation. The purpose was to decrease the applied voltage for changing the wettability by tuning the internal parameters of the structure.

The method describes the surface property evolution in the function of different parameters like the roughness degree, droplet dimensions, and surface tension of the fluid, and offers the optimization solution for the CP layer in order to present a specific hydrophobic/hydrophilic behavior.

The illustrated dependences of the wettability properties on different internal and external parameters allow us to consider the available CP variant and its working conditions for a specific application and, at the same time, points out the mutual influence of these internal and external exploiting parameters. E.g., the composed CP porous film of poly(3-hexylthiophene) (P3HT) + [6,6]-phenyl-C61-butyricacid-methyl-ester (PCBM) changes its wettability at voltages of up to 26 V, but if the doping ions are considered and the roughness geometry is modified, the applied voltage decreases twice.

An important fact illustrated by our analysis method is that the contact angle evolution, when the polymer wettability commutes, is driven mainly by the applied voltage, but the CA dependence on this voltage is strongly dependent on another structural parameter, which can be changed for wettability control.

Another important fact is that our method is able to illustrate that polymer wettability does not commute for specific sets of parameters. The values of these parameters can be determined and correlated with the polymer’s behavior in specific applications.

The applied method also illustrates that the thin film effect and supplementary dopants (ex-situ dopants added after the implicit in-situ structural doping) generate a significant decrease in the voltage that is necessary for wettability commutation.

Consequently, the applied non-invasive simulation method offers the possibility of optimizing the parameters that characterize the synthesizing and operating processes of the conductive polymers with dopants, from the point of view of their surface interactions with the fluid media. A significant variation (up to 30%) of the contact angle was demonstrated by a proper correlation of the structural parameters and the applied stimuli for wettability commutation.

## Figures and Tables

**Table 1:** Method of analysis applied for the determination of wetting properties for the considered conductive polymers, and comparison of the results and methods indicated in the literature with those of our method.

Conductive Polymer + Dopant	CA (Hydrophiic/Hydrophobic)	Control Parameters	Applications	Method of Determination/Source
**Ppy(DBS)** *	*Reported in literature:*			
	105° to 56°	Potential applied changes from positive (oxidative) to negative (reductive); Droplet nature (oil, water, organic droplet (CH_2_Cl_2_)); Droplet size; CP film thickness; Volatge decreases at low values (−0.9~0.6 V)	Ultra-low voltage digital microfluidics applications	Experimental (CA measured with a goniometer system)
	Hydrophobic ―> hydrophilic when voltage decreases (3.6~0.5 V); Induced non-reversible wettability			
		Thin film (1 μm polymer thickness); No supplementary cations/anion dopants in CP		Xu et al. [19]; Mugele et al. [22]
	*Our method:*			
	104° ―> 70° (20 V ―> 3.1 V; when droplet radius *a* increases); reversible;	Droplet radius *a* Surface roughness *r* Surface tension of the fluid *γ* Nature of the polymerizable core In-situ dopants nature Ex-situ dopants nature	similar	Theoretical (CSF model improved by Jasper et al. [5]) Simulation (VOF model—HFSS; CSF model Brackbill et al. [29]—HFSS, Mathcad)
	70° ―> 101° (20 V ―> 0; when surface roughness *r* increases); reversible;			
		Thin film (1000 nm polymer thickness); Cations/anion dopants in CP;		
	77° ―> 120° (20 V ―> 0; when surface tension of the fluid *γ* decreases); reversible;			
		Intervals of voltage and set of parameters for which wettability do not commute.		
**P3HT:PCBM**	*Reported in literature:*			
	144.7° to 25°	Concentration of solution for preparing the porous film; Surface roughness	Porous film; Organic photovoltaics; Gas sensing, adsorption, catalysis, porous electrodes, tissue engineering, biomaterials	Experimental (CA measured with a CA system JC2000C, Shanghai Zhongchen Technology Co. Ltd., Shanghai, China); SEM (JEOL JSM-7500, Tokyo, Japan)
	Hydrophobic ―> hydrophilic when voltage increases (2–26 V); Reversible wettability with hysteresis			
		Thin film (20 μm polymer thickness, due to porosities); No supplementary cations/anion dopants in CP		
				Teng et al. [7]
	*Our method:*			
	120° ―> 70° (20 V ―> 1.6 V; when droplet radius *a* increases); reversible;	Droplet radius Surface roughness Surface tension of the fluid Nature of the polymerizable core In-situ dopants nature Ex-situ dopants nature	similar	Theoretical (CSF model improved by Jasper et al. [5]) Simulation (VOF model—HFSS; CSF model Brackbill et al. [29]—HFSS, Mathcad)
	70° ―> 118° (20 V ―> 0; when surface roughness *r* increases); reversible;			
		Thin film (1000 nm polymer thickness); Cations/anion dopants in CP;		
	83° ―> 120° (20 V ―> 0; when surface tension of the fluid *γ* decreases); reversible;			
		Intervals of voltage and set of parameters for which wettability do not commute.		
**PEDOT:PSS-PEI**	*Reported in literature:*			
	120° to 70°	Surface tension of the liquid droplet, *γ*; Gating of capillary wetting; Voltage decreases (10–20 V)	Nonwoven textile; Sensing, Controllable coatings	Experimental (CA measured with AST VCA-Optima system); SEM; Theoretical (Cassie and Baxter eq.)
	Hydrophobic ―> hydrophilic when voltage increases from 4 to 100 V; Reversible electrowetting			
		Thin film (hundred of nm polymer thickness); No supplementary cations/anion dopants in CP		
				Bhat et al. [23]
	*Our method:*			
	120° ―> 70° (20 V ―> 0.6 V; when droplet radius *a* increases); reversible;	Droplet radius Surface roughness Surface tension of the fluid Nature of the polymerizable core In-situ dopants nature Ex-situ dopants nature	similar	Theoretical (CSF model improved by Jasper et al. [5]) Simulation (VOF model—HFSS; CSF model Brackbill et al. [29]—HFSS, Mathcad)
	74° ―> 120° (20 V ―> 0; when surface roughness *r* increases); reversible;			
		Thin film (1000 nm polymer thickness); Cations/anion dopants in CP;		
	81° ―> 120° (20 V ―> 0.1 V; when surface tension of the fluid *γ* decreases); reversible;			
		Intervals of voltage and set of parameters for which wettability do not commute.		

* The color of letters in the polymer name is the same as the color of the surface plot corresponding to the same polymer, in the 3D graphs.

## Data Availability

Not applicable.
